# Targeted gene inactivation in *Clostridium phytofermentans* shows that cellulose degradation requires the family 9 hydrolase Cphy3367

**DOI:** 10.1111/j.1365-2958.2009.06890.x

**Published:** 2009-10-12

**Authors:** Andrew C Tolonen, Amanda C Chilaka, George M Church

**Affiliations:** 1 Department of Genetics, Harvard Medical SchoolBoston, MA 02115 USA; 2 Department of Biology, Northeastern UniversityBoston, MA 02115 USA

## Abstract

Microbial cellulose degradation is a central part of the global carbon cycle and has great potential for the development of inexpensive, carbon-neutral biofuels from non-food crops. *Clostridium phytofermentans* has a repertoire of 108 putative glycoside hydrolases to break down cellulose and hemicellulose into sugars, which this organism then ferments primarily to ethanol. An understanding of cellulose degradation at the molecular level requires learning the different roles of these hydrolases. In this study, we show that interspecific conjugation with *Escherichia coli* can be used to transfer a plasmid into *C. phytofermentans* that has a resistance marker, an origin of replication that can be selectively lost, and a designed group II intron for efficient, targeted chromosomal insertions without selection. We applied these methods to disrupt the c*phy3367* gene, which encodes the sole family 9 glycoside hydrolase (GH9) in the *C. phytofermentans* genome. The GH9-deficient strain grew normally on some carbon sources such as glucose, but had lost the ability to degrade cellulose. Although *C. phytofermentans* upregulates the expression of numerous enzymes to break down cellulose, this process thus relies upon a single, key hydrolase, Cphy3367.

## Introduction

Cellulose is the most abundant renewable, biological energy source on earth (Leschine, 1995), and its degradation is a key part of the global carbon cycle. Cellulose has tremendous potential as a biofuel feedstock; more than a billion tons of lignocellulosic plant biomass could be used each year for liquid biofuels in North America (Perlack *et al.*, 2005). The major challenge of producing biofuel from cellulose is the recalcitrance of cellulosic fibres to break down into sugars. The current cost of converting cellulosic biomass to sugar doubles the carbohydrate purchase cost, nullifying the advantage of biomass relative to corn (Lynd *et al.*, 2008). Microbes both that secrete enzymes to break down cellulosic biomass and that ferment the resulting saccharides can make cellulosic biofuels more efficient by obviating feedstock pre-treatment and raising conversion efficiencies. However, the development of robust microbial strains for conversion of biomass to fuels has been hindered by our inability to genetically manipulate these organisms.

**Fig. 1:**
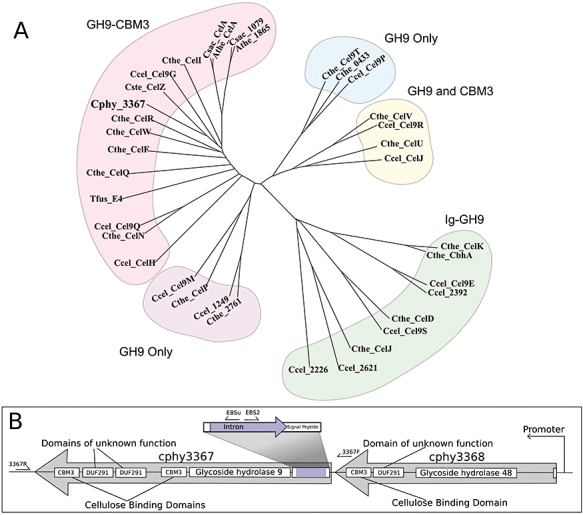
A. Unrooted dendrogram of family 9 glycoside hydrolase amino acid sequences shows that Cphy3367 belongs to the subfamily in which a CBM potentiates the adjacent hydrolytic domain. Abbreviations: Cphy, *Clostridium phytofermentans*; Cthe, *Clostridium thermocellum*; Cste, *Clostridium stercorarium*; Ccel, *Clostridium cellulolyticum*; Tfus, *Thermomonospora fusca*; Csac, *Caldicellulosiruptor saccharolyticus*; Athe, *Anaerocellum thermophilum*. B. Diagram of the c*phy3367–cphy3368* putative operon showing the protein domains, the site of intron insertion in *cphy3367* and the location of primers used to map the intron insertion by PCR.

*Clostridium phytofermentans*, a mesophilic anaerobe isolated from forest soil (Warnick *et al.*, 2002), is an ideal microbe to study the direct conversion of cellulosic biomass to ethanol. It grows on both of the two main components of plant biomass, cellulose and hemicellulose, by secreting enzymes to cleave these polysaccharides and then fermenting the resulting hexose (glucose, galactose, mannose) and pentose (xylose, arabinose) sugars to ethanol. Among clostridial genomes sequenced to date, *C. phytofermentans* has the highest number of genes encoding enzymes for the modification and breakdown of complex carbohydrates. It contains genes for 161 carbohydrate-active enzymes (CAZy), which include 108 glycoside hydrolases spread across 39 families (Cantarel *et al.*, 2009). This abundance of hydrolases highlights how cellulosic biomass is a complex substrate whose degradation likely requires the concerted action of many enzymes. Cellulolytic clostridia usually have numerous genes encoding family 9 glycoside hydrolases (GH9): the *Clostridium thermocellum* ATCC 27405 genome has 16 GH9 genes, *Clostridium cellulolyticum* H10 has 13 GH9 genes, and *Clostridium cellulovorans* has five GH9 genes. In contrast, *C. phytofermentans* has only a single GH9-encoding gene, *cphy3367*. GH9 proteins are all β-1,4-glucanases, but members of the GH9 family may have different substrate specificities and end-products (Arai *et al.*, 2006). There are subfamilies of GH9 proteins with different molecular architectures (Fig. 1A). Cphy3367 belongs to the subfamily, called theme B (Gilad *et al.*, 2003), in which the hydrolytic module is fused to a family 3 carbohydrate-binding module (CBM) (Fig. 1B).

Cellulolytic bacteria secrete a battery of glycoside hydrolases to depolymerize cellulosic biomass. The different roles of these hydrolases could be uncovered by targeted gene inactivation. Although cellulolytic clostridia have been studied for decades, targeted mutagenesis in these organisms has remained challenging, likely due to highly active DNases and inefficient homologous recombination. Previously, cellulolysis-deficient strains of *C. cellulolyticum* were isolated by spontaneous mutation of the scaffoldin gene, which anchors the cellulolytic enzymes to the cell surface (Maamar *et al.*, 2003; 2004), and by reduction of CelF48 expression with antisense-RNA (Perret *et al.*, 2004). In this study, we developed a general system for targeted, chromosomal gene inactivation in *C. phytofermentans.* Efficient transfer of DNA into *C. phytofermentans* is achieved by conjugation with *Escherichia coli* expressing the broad-host-range RP4 conjugal apparatus. Conjugation has been used transfer plasmids to several mesophilic clostridia including *Clostridium acetobutylicum* (Williams *et al.*, 1990), *C. perfringens* (Lyras and Rood, 1998), *C. cellulolyticum* (Jennert *et al.*, 2000) and *C. difficile* (Purdy *et al.*, 2002). To make targeted chromosomal insertions in *C. phytofermentans*, we modified a Gram-positive conjugal shuttle vector (Trieu-Cuot *et al.*, 1991) to contain a strong *C. phytofermentan*s promoter driving expression of a group II intron that was designed to inactivate *cphy3367.*

Group II introns are catalytic RNAs that insert into dsDNA in a site-specific manner called retrohoming (reviewed by Lambowitz and Zimmerly, 2004). The intron used in this study is derived from the *Lactococcus lactis* Ll.LtrB group II intron (Karberg *et al.*, 2001). It consists of a 0.9 kb Ll.LtrB-deltaORF intron flanked by short exon sequences and a downstream *ltrA* gene that encodes a protein with endonuclease and reverse transcriptase activity (Guo *et al.*, 2000). The intron inserts into the genome by splicing into a 13–16 bp DNA recognition sequence. The LtrA protein then cleaves the opposite DNA strand and uses the 3′ DNA as a primer to reverse transcribe the inserted intron RNA. Because the DNA binding specificity of the group II intron is conferred by short sequences, the intron can be easily modified to integrate into a desired DNA target site. Group II introns can be made to insert into virtually any DNA sequence with frequencies in *E. coli* of 0.1–22% (Karberg *et al.*, 2001). In theory, group II introns can function in any bacterial taxa into which plasmid DNA can be delivered because intron insertion does not require host-supplied factors. Group II introns have been used to make mutations in both Gram-negative bacteria (Yao and Lambowitz, 2007) and Gram-positive bacteria, including clostridia (Heap *et al.*, 2007). Previous experiments in the Gram-positive *Staphylococcus aureus* found that this mobile group II intron made targeted gene disruptions in 37–100% of colonies (Yao *et al.*, 2006).

Broadly, our goal is to dissect the set of cellulolytic enzymes in *C. phytofermentans* in order to identify key genes for the degradation of cellulosic biomass. The *C. phytofermentans* genome differs from other cellulolytic clostridia in encoding only a single GH9, Cphy3367. In this study, we disrupted the *cphy3367* gene with a targeted group II intron, which was delivered into *C. phytofermentans* by conjugation with *E. coli.* Inactivation of *cphy3367* resulted in a strain that grew normally on glucose, cellobiose and hemicellulose, but had lost the ability to degrade crystalline cellulose. mRNA expression supports that *cphy3367* is among the most highly upregulated CAZy genes during growth on cellulose. These findings reveal a central role played by Cphy3367 in cellulose degradation. Generally, these results show that targeted gene inactivation can be used to identify key enzymes for cellulose degradation in *C. phytofermentans.*

## Results

### A group II intron plasmid for use with *C. phytofermentans*

**Fig. 2:**
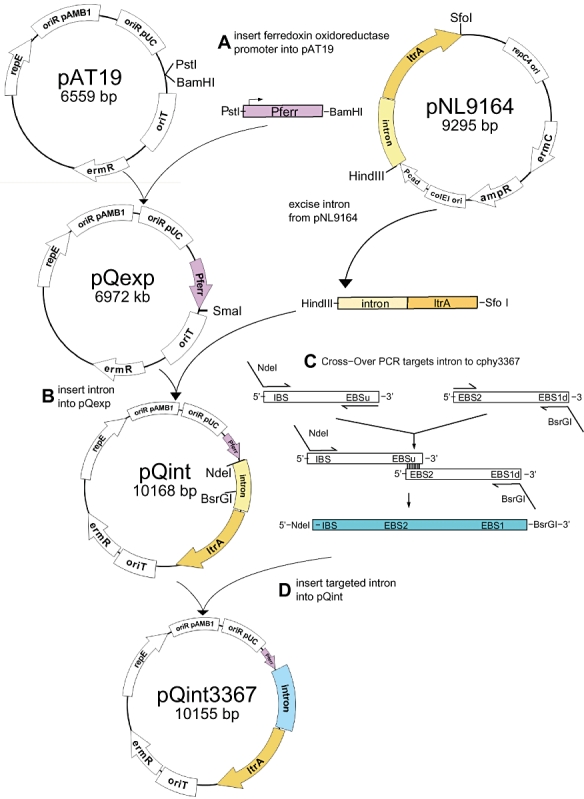
Construction of a plasmid for inactivation of *cphy3367* with a group II intron. A. The *C. phytofermentans* pyruvate ferrodoxin oxidoreductase promoter (Pferr) was inserted into pAT19 to make pQexp. B. The intron cassette from pNL9164 was inserted downstream of Pferr in pQexp to make pQint. C. The intron cassette was retargeted to *cphy3367* by two-step, cross-over PCR. D. The retargeted intron was inserted into pQint to make pQint3367. Plasmid diagrams show restriction sites used in cloning.

The plasmid we developed for gene inactivation in *C. phytofermentans* is derived from pAT19 (Trieu-Cuot *et al.*, 1991). This plasmid has an RP4 conjugal origin of transfer, origins of replication both for *E. coli* (pUC) and for Gram-positive bacteria (*Enterococcus* pAMβ1) and an erythromycin resistance gene from *Streptococcus pneumoniae* Tn*1545* that functions in both Gram-negative and Gram-positive bacteria. This plasmid was modified to disrupt *cphy3367* with a group II intron in three steps: addition of a strong *C. phytofermentans* promoter to pAT19, insertion of the intron (Ll.LtrB-deltaORF and *ltrA* gene) downstream of the *C. phytofermentans* promoter, and targeting the intron to *cphy3367*. In the first step, the promoter from *C. phytofermentans* pyruvate ferredoxin oxidoreductase (*cphy3558*) was cloned into pAT19 to yield pQexp (Fig. 2A). Pyruvate ferredoxin oxidoreductase functions in central carbon metabolism to couple the reduction of ferredoxin with the oxidative decarboxylation of pyruvate to acetyl-CoA and CO_2_. Genome-wide studies of *C. phytofermentans* mRNA (J. Blanchard, pers. comm.) support that *cphy3558* is among the most highly expressed genes in the genome on all tested carbon sources. The gene upstream of *cphy3558* in the genome is transcribed in the opposite direction, showing that *cphy3558* is transcribed from its own promoter. The high, constitutive expression of *cphy3558* supports that this promoter can be used in pQexp for strong expression of the group II intron, or other genes, in *C. phytofermentans* under diverse physiological conditions.

The group II intron and *ltrA* gene from pNL9164 (Yao *et al.*, 2006) were inserted into pQexp downstream of the *cphy3558* promoter to produce pQint (Fig. 2B). The DNA binding region of the intron in pQint was retargeted to *cphy3367* by a two-step, cross-over PCR (Fig. 2C). An intron insertion site in *cphy3367* was selected that was both in the antisense orientation and near the start codon (Fig. 1B). An antisense site was used because introns integrated in the sense orientation can splice out from the RNA transcript *in vivo* with the help of LtrA (Yao *et al.*, 2006). An antisense insertion in *cphy3367* would yield an unconditional disruption, which was predicted to grow normally on media containing a simple carbon source such as glucose that does not require cleavage by a β-1,4-glucanase. The antisense insertion site nearest to the start codon was used in order to have the highest probability of disrupting *cphy3367* expression. This site is 37 bp downstream of the predicted start codon and resides in the putative secretion signal sequence (other potential sites in Supporting information S1). As *cphy3367* is the downstream member of a putative two gene operon (Fig. 1B), disruption of *cphy3367* should not result in polar transcriptional effects. The retargeted intron fragment was inserted into pQint to make pQint3367 (Fig. 2D)*.* The complete sequences of pQexp, pQint and pQint3367 are in Supporting information S2.

### DNA is efficiently transferred to *C. phytofermentans* by conjugation with *E. coli*

pQint3367 was introduced into *C. phytofermentans* ISDg (ATCC 700394) by conjugal transfer from *E. coli*. The conjugal donor was *E. coli* strain 1100-2 (Bandrin *et al.*, 1983) containing pRK24 (Meyer *et al.*, 1977), the plasmid encoding the RP4 conjugal apparatus, and pQint3367. Cells were mated on solid G2 medium overnight. Following mating, *C. phytofermentans* transconjugants were isolated by selecting against both *E. coli* and *C. phytofermentans* cells that did not receive pQint3367. As *C. phytofermentans*, but not *E. coli* 1100-2, is naturally resistant to nalidixic acid and trimethoprim, these antibiotics were used to prevent growth of *E. coli* 1100-2 after mating. If other *E. coli* strains that are less sensitive to these antibiotics are used for matings, additional steps may be needed to isolate pure *C. phytofermentans* colonies. For example, if plates are supplemented with X-gal and IPTG, *lacZ*+*E. coli* strains will form blue colonies whereas *C. phytofermentans* colonies will be white. Alternatively, liquid cultures can be inoculated with phage T7 before plating to selectively remove *E. coli* (Tolonen *et al.*, 2006). Relative to *C. phytofermentans* colony counts on plates lacking erythromycin, colony counts on plates containing erythromycin showed that the efficiency of plasmid transfer to *C. phytofermentans* was ∼1 transconjugant per 10^6^ recipient cells. This transfer rate is typically efficient enough to isolate 10–20 *C. phytofermentans* transconjugants per plate. In this study, 10 independent transconjugants were isolated, hereafter called AT02-1 to AT02-10. When erythromycin-resistant *C. phytofermentans* colonies were picked, the absence of residual *E. coli* cells was confirmed by plating an aliquot on solid LB medium and incubating aerobically at 37°C overnight.

### Genomic intron insertions in *C. phytofermentans* are accurate and specific

**Fig. 3:**
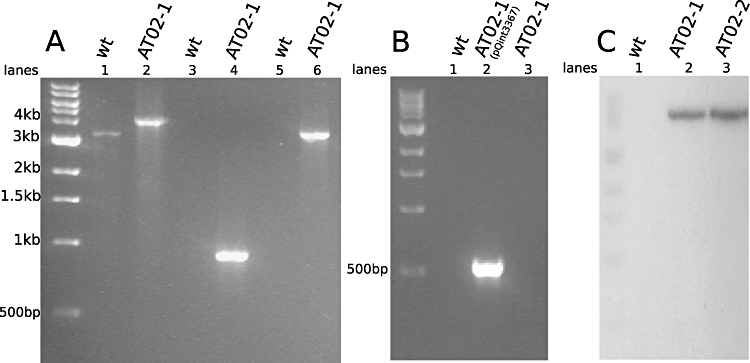
The intron insertion in c*phy3367* of AT02 is both accurate and specific. A. PCR of the *cphy3367* locus in wild-type and AT02-1 strains. The c*phy3367* gene in AT02-1 contains a 900 bp insertion relative to wild type (lanes 1 and 2). Primers to amplify the genome–intron junctions yield PCR products in AT02-1, but not wild type, for both the 5′ junction (lanes 3 and 4) and the 3′ junction (lanes 5 and 6). B. PCR of the erm^R^ gene from pQint3367 shows acquisition of the plasmid in AT02-1 following conjugation (lanes 1 and 2) and its loss by plasmid curing (lane 3). C. Southern blot probed with a ^32^P-labelled intron probe reveals no band in wild type (lane 1) and a single intron insertion in two independent transconjugants, AT02-1 and AT02-2 (lanes 2 and 3). The single band shown in the AT02 lanes was the only one visible on the blot (100 bp to 10 kb).

The presence of pQint3367 was examined in *C. phytofermentans* transconjugants by PCR amplification of the erm^R^ gene. The erm^R^ PCR product was absent in wild-type *C. phytofermentans* cultures, but was present in AT02-1 to AT02-10. The expected intron insertion, 37 bp downstream of the start codon *cphy3367* in the antisense direction (Fig. 1B), was confirmed by PCR mapping of the *cphy3367* locus in the AT02 isolates. PCR of the *cphy3367* coding region yielded a 3.1 kb product in wild type and a 4 kb product in AT02-1, supporting the expected 0.9 kb intron insertion in *cphy3367* (Fig. 3A, lanes 1 and 2). PCR of the 5′ and the 3′ intron–genome junction regions using one primer in the genome and the other in the intron (3367_F/Ebs2_3367 for the 5′ junction and 3367_R/Ebs_univ for the 3′ junction) resulted in products in AT02-1 (Fig. 3A, lanes 4 and 6), but not wild type (Fig. 3A, lanes 3 and 5). These PCR products were sequenced to confirm that AT02-1 bears the expected intron insertion in *cphy3367* (Fig. S1). Similarly, PCR screening of the other nine AT02 isolates confirmed the correct insertion in *cphy3367*, supporting that the Ll.LtrB group II intron is efficient and accurate in *C. phytofermentans*.

Once the correct intron insertion in *cphy3367* had been confirmed, the loss of pQint3367 was induced in AT02-1 and AT02-2 by diluting cultures 1:100 in GS2 (−erm) medium and growing to late log phase (OD_600_ 1.0) through five serial transfers. AT02-1 was then plated on solid GS2 (−erm) medium and 10 colonies were tested by PCR, both for the presence of the erm^R^ gene in pQint3367 and for the retention of an intron insertion in *cphy3367* with primers 3367_F and Ebs2_3367. Transfer in medium lacking erythromycin resulted in rapid loss of pQint3367 (Fig. 3B). In total, 8 of 10 colonies had lost pQint3367, while all 10 had retained the intron insertion in *cphy3367* (Fig. S2A). Further, plating on GS2 medium containing erythromycin showed that strains that had lost pQint3367 were erythromycin-sensitive (Fig. S2B). After an additional five transfers in the absence of erythromycin selection, it was again confirmed that all 10 AT02-1 cultures still retained the genomic intron insertion in *cphy3367*. The pQint plasmid can thus be used to make genomic insertions in *C. phytofermentans* that are stable in the absence of selection. Because the pQint plasmid can be efficiently lost in the absence of selection and the intron does not contain an antibiotic resistance gene, multiple genes in the same *C. phytofermentans* strain can be inactivated by sequentially introducing pQint plasmids targeted to different genes.

Southern blotting showed that AT02-1 and AT02-2 do not harbour any additional intron insertions elsewhere in the genome (Fig. 3C). The Southern blot showed no bands in wild type (Fig. 3C, lane 1) and a single band in both transconjugants, AT02-1 and AT02-2, at the expected size of 5855 bp (Fig. 3C, lanes 2 and 3). A Southern blot of AT02-1 prior to loss of pQint3367 showed a second band of 7799 bp due to the plasmid-born intron. The Ll.LtrB group II intron can thus be used for targeted gene disruptions in *C. phytofermentans* without making additional, unintended genomic insertions.

### AT02-1 grows normally on glucose, cellobiose and hemicellulose, but is unable to degrade filter paper cellulose

**Fig. 4:**
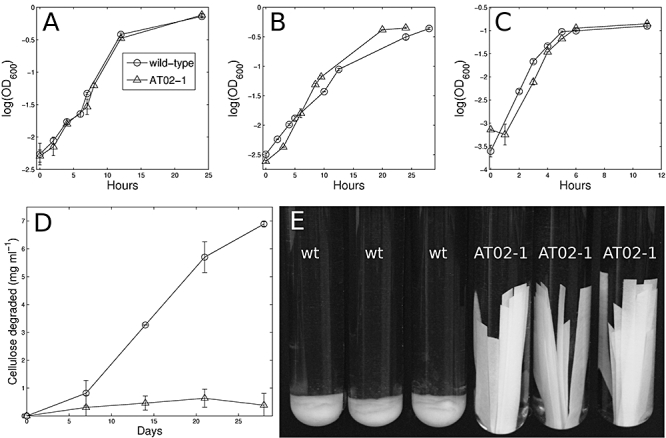
A–C. Strain with disruption of c*phy3367* (AT02-1) had similar growth rates as wild type on glucose (A), cellobiose (B) and hemicellulose xylan (C). Growth curves are means of triplicate cultures. Error bars show one standard deviation and are smaller than the symbols where not apparent. D. Disruption of c*phy3367* results in inability to degrade filter paper cellulose. Cellulose degradation was measured as dry mass of cellulose remaining in culture. E. After 4 weeks, cellulose strips in wild-type (wt) tubes had broken down while strips in AT02-1 cultures appeared unchanged.

Growth rates (OD_600_) of AT02-1 and wild-type strains were compared in GS2 media containing either glucose, cellobiose or hemicellulose xylan as the sole carbon source. As expected, wild type and AT02-1 showed similar growth rates on glucose (Fig. 4A). The comparable growth rates of wild type and AT02-1 on cellobiose (Fig. 4B) supports that the glucanase activity of the Cphy3367 protein is not required to break the 1,4-β bond between glucose subunits of cellobiose. Further, wild type and AT02-1 grew at similar rates on hemicellulose (Fig. 4C). The hemicellulose used in this study was birchwood xylan, which is a polysaccharide of 89.3% xylose, 1% arabinose, 1.4% glucose and 8.3% anhydrouronic acid (Kormelink *et al.*, 1993). Although this assay does not establish whether Cphy3367 is active on β-1,4-d-xylose bonds, the robust growth of AT02-1 on hemicellulose shows that Cphy3367 is not required for *C. phytofermentans* to metabolize hemicellulose.

The cellulose degradation rates of AT02-1 and wild type were measured in cultures of GS2 medium with filter paper cellulose as the sole carbon source. We found that wild type consumed cellulose at a mean rate of 0.24 mg ml^−1^ day^−1^, while cellulose consumption by AT02-1 was negligible (Fig. 4D). The composition of the cellulose in wild-type and AT02-1 cultures was visibly different after a few days. Following the 4-week experiment, the remaining cellulose in wild-type cultures was a viscous pulp, while the cellulose strips in AT02-1 cultures remained unchanged (Fig. 4E). Disruption of *cphy3367* thus renders *C. phytofermentans* unable to degrade filter paper cellulose.

### Wild-type and AT02-1 expression of *cphy3367* and other genes on cellulosic carbon sources

**Fig. 5:**
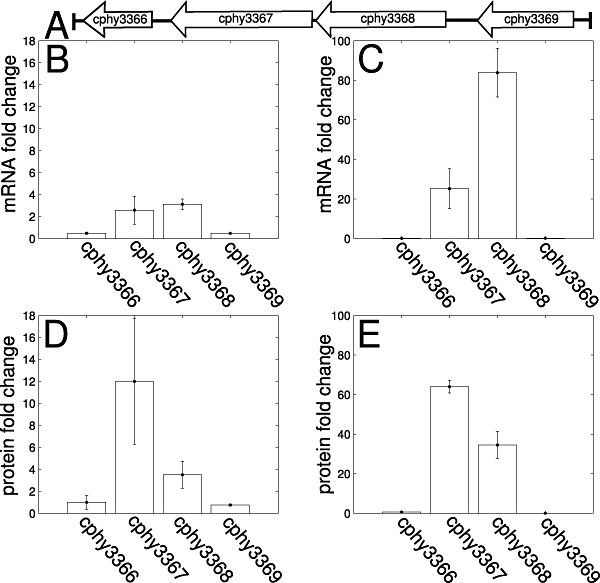
Expression changes of *cphy3367* and surrounding genes (A) in the genome on different carbon sources. Wild-type mRNA (B and C) and protein (D and E) expression of *cphy3367* and *cphy3368* increased in hemicellulose (B and D) and cellulose (C and E) cultures compared with glucose while expression of the surrounding genes did not increase. The mRNA expression was measured by qRT-PCR. Bars show mean fold change calculated as 2^−ΔΔCt^. Protein expression was quantified by mass spectrometry. Bars show mean number of peptides detected from each protein in hemicellulose or cellulose cultures divided by the number of peptides detected in glucose cultures.

The inability of AT02-1 to degrade cellulose prompted us to examine the expression patterns of *cphy3367* and related genes. Initially, we compared the expression of *cphy3367* with those of surrounding genes in the genome to see which genes were coexpressed with *cphy3367*. The *cphy3367* gene is 76 bp downstream of *cphy3368*, a family 48 glycoside hydrolase, and is 341 bp upstream of *cphy3366*, a MoxR-like ATPase (Fig. 5A). Sequence analysis suggests that *cphy3366* is transcribed from its own promoter; disruption of *cphy3367* would thus not result in polar transcriptional effects. A putative promoter upstream of *cphy3368* led us to hypothesize that *cphy3367* is the second gene in a putative two gene operon (Fig. 1B). In support of this hypothesis, we found that in wild-type cells *cphy3367* and *cphy3368* mRNA transcripts are upregulated on hemicellulose (Fig. 5B) and cellulose (Fig. 5C) relative to expression on glucose, while the surrounding genes did not increase in expression. Similar expression changes were observed for the Cphy3366–Cphy3369 proteins on hemicellulose (Fig. 5D) and cellulose (Fig. 5E). The *cphy3367–cphy3368* putative operon is thus upregulated independent of the surrounding genes, which do not appear to be involved in acclimation to growth on cellulosic biomass. Further, the much higher upregulation of *cphy3367* on cellulose than on hemicellulose supports that this gene is more directly involved in the degradation of cellulose.

**Fig. 6:**
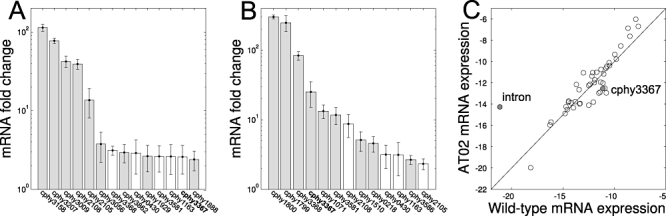
mRNA expression changes of genes encoding carbohydrate-active enzymes. A and B. Genes that were greater than twofold upregulated in wild-type cells grown on hemicellulose (A) and cellulose (B) relative to expression on glucose are shown. Genes that were also greater than twofold upregulated on hemicellulose relative to cellulose in (A) or vice versa in (B) are shaded. C. The mRNA expression of genes for carbohydrate-active enzymes was similar in wild-type and AT02-1 strains on hemicellulose, supporting that disruption of *cphy3367* does not result in widespread changes in the expression of other enzymes. The mRNA expression was measured by qRT-PCR. Bars in (A) and (B) show mean fold change calculated as 2^−ΔΔCt^; circles in (C) show −ΔCt. Errors show one standard deviation. Expression of *cphy3367* and the intron integrated in *cphy3367* in AT02-1 are shaded in (C).

The mRNA expression of *cphy3367* on cellulosic carbon sources was also compared with the expression of 39 other CAZy genes by qRT-PCR. The set of CAZy genes we examined included 33 glycoside hydrolases spread across 20 families, two carbohydrate esterases, three glycosyl transferases and two polysaccharide lyases (see Supporting information S3 for a complete list of genes). Among these 40 CAZy genes, 14 genes were greater than twofold upregulated on hemicellulose (Fig. 6A) and 13 genes were upregulated on cellulose (Fig. 6B) relative to their expression during growth on glucose. Moreover, there were widespread differences in the mRNA expression of CAZy genes between hemicellulose and cellulose (shaded bars in Fig. 6A and B). *C. phytofermentans* can thus differentiate between the components of cellulosic biomass and respond by transcriptionally altering the set of CAZy genes that are expressed. Only three other CAZy enzymes (*cphy1799*, *cphy1800*, *cphy3368*) were more highly upregulated than *cphy3367* on cellulose (Fig. 6B), while a dozen CAZy genes were more highly upregulated than *cphy3367* on hemicellulose (Fig. 6A), further supporting that Cphy3367 is more directly involved in the breakdown of cellulose than hemicellulose. As the acclimation to growth on cellulosic carbon sources in *C. phytofermentans* involves the increased expression of numerous CAZy genes, the breakdown of cellulosic substrates likely requires the concerted action of additional enzymes to Cphy3367.

The mRNA expression of CAZy genes in wild-type and AT02-1 cells growing on hemicellulose was examined to determine if disruption of *cphy3367* affected the expression of additional genes. While *cphy3367* expression was elevated on hemicellulose (Fig. 5B), the mRNA expression of CAZy genes in AT02-1 and wild-type cells was highly similar (Fig. 6C). Proper expression of *cphy3367* is thus not required for the induction of other CAZy genes, supporting that the inability of strain AT02-1 to degrade cellulose is specifically due to loss of the Cphy3367 protein. The expression of *cphy3366*, the gene downstream of *cphy3367* in the genome, was moderately lower in AT02-1 than in wild type when growing on hemicellulose, but these changes were less than twofold. Further, we found that mRNA expression of the intron was similar to *cphy3367* in AT02-1, but was not detected in wild-type cells (Fig. 6C).

## Discussion

Although the *C. phytofermentans* genome encodes over a hundred glycoside hydrolases and many of these genes are upregulated on cellulosic carbon sources, this study shows that the inactivation of a single hydrolase, Cphy3367, results in an inability to degrade cellulose. To enable the genetic analysis of cellulose degradation by *C. phytofermentans*, the initial contribution of this study was to develop a system to make targeted insertions in the *C. phytofermentans* chromosome*.* We showed that interspecific conjugation with *E. coli* can be used to reliably transfer foreign DNA into *C. phytofermentans*. The plasmid we transferred to *C. phytofermentans* established that the erm^R^ gene from *S. pneumoniae* Tn*1545* gives high erythromycin resistance in this organism. Further, the *Enterococcus* pAMβ1 origin of replication replicates stably in *C. phytofermentans* under antibiotic selection, but can be easily cured from strains in the absence of selection. Finally, the *L. lactis* Ll.LtrB group II intron makes accurate (Fig. 3A) and specific (Fig. 3C) chromosomal insertions in *C. phytofermentans*. We found that when the strong *C. phytofermentans cphy3558* promoter is used to drive expression of the intron, it inserts into the genome with such a high efficiency that mutants can be easily isolated without selecting for integration. As the intron insertions are stable in the absence of selection, the intron need not contain a resistance gene. Once the plasmid is cured from *C. phytofermentans*, no antibiotic resistance genes remain. Multiple intron insertions can thus be made in the same strain without needing independent resistance markers. This is particularly helpful because so few resistance markers are known to function in clostridia. It is, however, possible that intron insertions in certain regions of the genome might occur with lower efficiency such that a direct selection for integration would be advantageous. Group II introns have been modified to contain Retrotransposition-Activated Markers (RAM) to positively select for integration (Zhong *et al.*, 2003). RAM consist of an antibiotic resistance gene that is interrupted by a group I intron. When the group II intron integrates, the group I intron is excised from the resistance gene. RAM have been used to positively select for intron insertions in other clostridia (Heap *et al.*, 2007) and would likely be useful in *C. phytofermentans* to obtain strains with intron insertions in low-efficiency positions in the genome.

We applied these methods to study the genetic basis of cellulose degradation by disrupting *cphy3367*, encoding the only GH9 in *C. phytofermentans*. The GH9-deficient strain, AT02-1, grows normally on glucose, cellobiose and hemicellulose (Fig. 4A–C) but has lost the ability to degrade crystalline cellulose (Fig. 4D and E). Several lines of evidence support that this phenotype resulted specifically from the inactivation of Cphy3367. AT02-1 did not have any additional intron insertions (Fig. 3C). The gene expression patterns of *cphy3367* and surrounding genes (Fig. 5) support that *cphy3367* is the downstream member of a two-gene operon such that inactivation of *cphy3367* would not result in polar transcriptional effects. Finally, the expression of CAZy genes in wild type and AT02-1 appears similar, supporting that disruption of *cphy3367* does not result in altered expression of other genes for the breakdown of cellulose.

In contrast to other clostridia such as *C. thermocellum*, *C. cellulolyticum* and *C. cellulovoran*s, the glycoside hydrolases in *C. phytofermentans* do not appear to multimerize into an extracellular cellulosome. The *C. phytofermentans* genome does not appear to encode a cellulosomal scaffoldin to anchor the cellulases to the cell surface and the hydrolases do not have dockerin domains to attach to a scaffoldin. The inability of AT02-1 to degrade cellulose is unlikely to result from the disruption of an extracellular, cellulosome-like complex. Although *C*. *phytofermentans* is different from other cellulolytic clostridia in having only one family 9 hydrolase, numerous other *C. phytofermentan*s hydrolases are upregulated on hemicellulose and cellulose (Fig. 6), suggesting that the breakdown of cellulose requires the co-ordinated action of multiple enzymes.

**Fig. 7:**
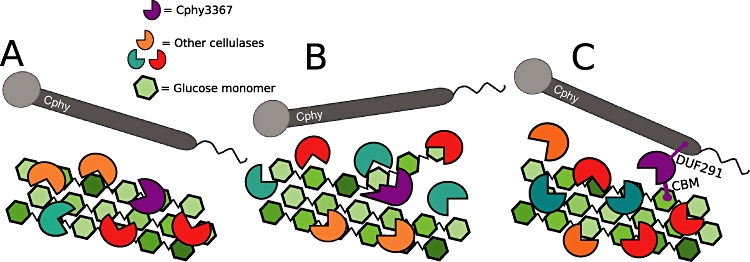
Non-exclusive models for the roles of Cphy3367 in cellulose degradation. A. The set of *C. phytofermentans* cellulolytic enzymes has members, such as Cphy3367, with key hydrolytic functions for which there is no redundancy. B. The CBM on Cphy3367 acts as a wedge that is required to free cellulose chains such that they can be cleaved by Cphy3367 and other cellulases. C. Attachment of the cell to the cellulosic substrate by Cphy3367 is required for efficient cellulose degradation. The Cphy3367 DUF291 domain binds to the cell and the CBM binds to cellulose.

How do we explain the critical dependence of *C. phytofermentans* upon Cphy3367 for cellulose degradation? There are well-studied members of the same GH9 subfamily as Cphy3367 (Fig. 1A). The protein with the highest sequence similarity to Cphy3367 in the NCBI database is *Clostridium stercorarium* CelZ, with which Cphy3367 shares 65% amino acid identity. Cphy3367 and CelZ have the same domain organization and the CelZ catalytic residues D84 and E447 (Riedel and Bronnenmeier, 1999) are conserved in Cphy3367 as D83 and E446. Degradation of crystalline cellulose is typically achieved by a synergy between endocellulases to liberate cellulose chains from the cellulose surface and exocellulases to cleave these chains into oligosaccharides. However, GH9 proteins in the subfamily with Cphy3367, such as *C. stercorarium* CelZ, are unusual in that they act both as endoglucanases and as exoglucanases (Jauris *et al.*, 1990). Purified *C. thermocellum* CelI is sufficient to solubilize filter paper *in vitro* (Gilad *et al.*, 2003). Our results build upon this finding by showing that Cphy3367 is required by *C. phytofermentans* to solubilize filter paper *in vivo*. Cphy3367 may have the central role in cellulose degradation, with other cellulases having accessory functions. Alternatively, some or all of the *C. phytofermentans* cellulases may have required non-redundant functions to break down cellulose such that inactivation of these genes would have similar phenotypes to AT02 (Fig. 7A). Future gene inactivation studies will reveal the relative contributions of other *C. phytofermentans* hydrolases to cellulose degradation.

Besides the GH9 hydrolytic module, Cphy3367 has additional domains that play important roles in cellulose degradation. Cphy3367 has two family 3 CBMs (CBM3): one adjacent to the hydrolytic domain and one at the C-terminus (Fig. 1B). CBMs promote polysaccharide degradation by bringing the enzyme into prolonged, close association with the surface of the carbohydrate. Although CBMs are generally non-catalytic, some CBMs have been shown to actively disrupt the polysaccharide structure (Din *et al.*, 1994). In the GH9 subfamily with Cphy3367, the CBM adjacent to the GH9 domain has been shown to potentiate the catalytic activity while the C-terminal CBM anchors the enzyme to the insoluble, cellulosic substrate (Sakon *et al.*, 1997). Both CBMs are required for the enzyme to have full activity on crystalline cellulose (Gilad *et al.*, 2003). It has been proposed that the hydrolase domain and adjacent CBM of CelZ structurally modify the substrate by freeing cellulose chains to make them accessible to other cellulases (Riedel and Bronnenmeier, 1999). In the *Hypocrea jecorina* Cel7A hydrolase, the CBM and the catalytic domain are proposed to function as a molecular machine (Mulakala and Reilly, 2005) where the CBM is a wedge to pry cellulose chains from the crystalline cellulose surface. The CBM then functions as a Brownian ratchet to pass the freed chain to the GH domain for cleavage. Similarly, *C. phytofermentans* may require Cphy3367 to free cellulose chains from the cellulose fibril for attack by other cellulases (Fig. 7B).

Cphy3367 also has two, tandem C-terminal domains of unknown function (Fig. 2B) that are alternatively called DUF291, hydrophilic domains (Pages *et al.*, 1999), or X2 modules (Mosbah *et al.*, 2000). In addition to being found in Cphy3367, DUF291 domains are found in Cphy2128 (GH26), Cphy3202 (GH5) and Cphy3368 (GH48). Homologous domains are found in the scaffoldin proteins of *C. cellulolyticum* (CipC), *C. cellulovorans* (CbpA) and *C. josui* (CipA) as well as the hydrolases CelY and CelZ from *C. stercorarium*. The CbpA DUF291 domains bind both cellulose and the cell wall (Kosugi *et al.*, 2004), and have thus been proposed both to promote cellulose degradation and to anchor the cellulosome to the cell surface. Similarly, the DUF291 domains of Cphy3367 may be critical to degrade cellulose or to attach Cphy3367 to the cell surface while the CBM domains bind Cphy3367 to the cellulosic substrate (Fig. 7C). The inability of AT02 to degrade cellulose could be due, at least in part, to an inability to retain the cell in close enough proximity to the cellulose substrate so that hydrolases can function efficiently.

Microbial cellulose degradation is of global ecological importance to recycle photosynthetically fixed carbon. Further, a mechanistic understanding of how microbes break down cellulose will facilitate the development of cellulosic biofuels as renewable, carbon-neutral alternatives to fossil fuels. This study demonstrates that we can now study the genetic basis of cellulose degradation using targeted chromosomal insertions in *C. phytofermentans*. If the intron is modified to contain a gene of interest, genomic insertion of the intron would allow stable expression of this gene on the chromosome. As these introns do not rely upon host-encoded factors for insertion, they may be generally applicable to the study of cellulolytic clostridia. In this study, we applied these introns for targeted disruption of a glycoside hydrolase gene, *cphy3367*. Among the many *C. phytofermentans* glycoside hydrolase genes that are upregulated during growth on cellulosic substrates, it is encouraging that the inactivation of a single gene had a strong effect upon cellulose degradation because it suggests that this process can be engineered using reverse genetics focusing on individual genes. Future genetic studies of *C. phytofermentans* will untangle the roles of additional hydrolases to provide a mechanistic understanding of cellulose degradation.

## Experimental procedures

### Culture conditions

*Escherichia coli* was cultured in LB medium supplemented with 50 μg ml^−1^ carbenicillin or 200 μg ml^−1^ erythromycin, as appropriate. *C. phytofermentans* was cultured anaerobically in GS2 (Johnson *et al.*, 1981) with either 3 g l^−1^ glucose, 3 g l^−1^ cellobiose, 3 g l^−1^ hemicellulose xylan or 12 g l^−1^ cellulose. The hemicellulose used in this study was birchwood hemicellulose xylan (Sigma X0502), which consists primarily of poly β-1,4-d-xylopyranose. Cellulose consisted of 0.5 × 5 cm strips of Whatman #1 filter paper (Cat 1001-090). Degradation of cellulose was quantified as the dry mass of cellulose remaining in culture. Cellulose was collected on filters by vacuum filtration, dried overnight at 65°C and weighed.

### Plasmid construction

The *cphy3558* promoter was inserted into pAT19 (Trieu-Cuot *et al.*, 1991) by PCR of the 400 bp region upstream of the *cphy3558* start codon using primers Pferr_F and Pferr_R and cloning this fragment between the PstI and BamHI sites in pAT19 such that the promoter was directed towards the BamHI site. In addition to containing a 5′ BamHI site, the primer used to amplify the downstream portion of the *cphy3558* promoter (Pferr_R) also contains an NdeI site just 3′ of the BamHI site. The NdeI site was used in a later step to target the intron to *cphy3367* and can be used to clone other genes for expression from the *cphy3558* promoter. The sequence of the *cphy3558* promoter in pQexp was verified using primers PferrSeq_F and PferrSeq_R. To insert the intron into pQexp, a fragment containing the intron and *ltrA* gene was excised from pNL9164 (Yao *et al.*, 2006) with HindIII and SfoI. The HindIII overhang was blunted using Klenow and the intron was cloned into pQexp at the SmaI site, which was directly downstream of the BamHI site used to clone the *cphy3558* promoter. The intron borders in pQint were verified by sequencing. The intron integration site was chosen by calculating all potential sites for intron insertion into the 5′ 750 bp of c*phy3367* using a probabilistic model (Perutka *et al.*, 2004) that is implemented on the Targetron website (http://www.sigma-aldrich.com). An antisense integration site 37 bp downstream from the start codon was chosen. PCR primers were designed to promote base pairing between *cphy3367* and the EBS1, EBS2, and gamma sequences of the intron RNA. The IBS1 and IBS2 sites in the 5′ exon of the intron were made complementary to the EBS1 and EBS2 sites for efficient RNA splicing. To retarget the intron to *cphy3367*, a 350 bp NdeI–BsrGI intron fragment containing the EBS, gamma and IBS sites was made by two-step cross-over PCR (Fig. 2C) using external primers Ibs_3367 and Ebs1D_3367 and internal primers Ebs2_3367 and Ebs_univ. The intron targeted to *cphy3367* was inserted between the NdeI and BsrGI of pQint to form pQint3367. The intron sequence in pQint3367 was sequence verified using primers 3367intron_F and 3367intron_R. The sequences of primers used in this study are shown in Table 1.

**Table 1:** PCR primers, plasmids and strains used in this study.

Primer/plasmid/strain	Primer sequence/plasmid features/strain genotype	Function/source
Primer		
Pferr_F	TGCATGCTGCAGTTTGGTCAACATTTAACCTC	Forward primer to clone *cphy3558* promoter
Pferr_R	ACGTACGGATCCCATATGGTTTGAATATCCTCCTT	Reverse primer to clone *cphy3558* promoter
PferrSeq_F	ATTAATGCAGCTGGCACGAC	Forward primer to sequence *cphy3558* promoter in pAT19
PferrSeq_R	CTGCAAGGCGATTAAGTTGG	Reverse primer to sequence *cphy3558* promoter in pAT19
Ebs_univ	CGAAATTAGAAACTTGCGTTCAGTAAAC	EBS universal primer to target pQint to *cphy3367*
Ibs_3367	AAAACATATGATAATTATCCTTAATGGACATCAGAGTGCGCCCAGATAGGGTG	IBS primer to target pQint to *cphy3367*
Ebs2_3367	TGAACGCAAGTTTCTAATTTCGGTTTCCATCCGATAGAGGAAAGTGTCT	EBS2 primer to target pQint to *cphy3367*
Ebs1d_3367	CAGATTGTACAAATGTGGTGATAACAGATAAGTCATCAGAAGTAACTTACCTTTCTTTGT	EBS1d primer to target pQint to *cphy3367*
3367intron_F	TTCGCCAGAAAACAAAAGAAA	Forward primer to sequence targeted intron in pQint3367
3367intron_R	ACTGTACCCCTTTGCCATGT	Reverse primer to sequence targteted intron in pQint3367
3367_F	ATTGGAACAAGGCAACTGCT	Forward primer upstream of *cphy3367*
3367_R	TAGCACTATTCGCGGACGAT	Reverse primer downstream of *cphy3367*
erm_F	TGGAACAGGTAAAGGGCATT	Forward primer for internal *ermR* fragment from pAT19
erm_R	GCGTGTTTCATTGCTTGATG	Reverse primer for internal *ermR* fragment from pAT19
Plasmid		
pAT19	*ermR*, pAMbeta1 origin, pUC origin, RP4 oriT	Trieu-Cuot *et al.* (1991)
pNL9164	Ll.LtrB-deltaORF intron, *ltrA*	Yao *et al.* (2006)
pRK24	RP4 conjugal genes, *tetR*, *ampR*	Meyer *et al.* (1977)
pQexp	pAT19 with *cphy3558* promoter	This study
pQint	pQexp with Ll.LtrB-deltaORF intron, *ltrA*	This study
pQint3367	pQint with intron targeted to *cphy3367*	This study
Strain		
*E. coli* 1100-2	*mcrA0, endA1, mcrB9999*	Bandrin *et al.* (1983)
*C. phytofermentans* ISDg	Wild type	ATCC 700394
*C. phytofermentans* AT02	*cphy3367*::intron	This study

Restriction enzyme sites in primers are underlined.

### Conjugation

The conjugal donor used in matings was *E. coli* strain 1100-2 (Bandrin *et al.*, 1983) transformed with the RP4 conjugal plasmid pRK24 (Meyer *et al.*, 1977) and pQint3367. To mate *E. coli* and *C. phytofermentans*, a 10 ml culture of the *E. coli* donor was grown overnight in LB medium containing 50 μg ml^−1^ carbenicillin to select for pRK24 and 200 μg ml^−1^ erythromycin to select for pQint3367. The culture was washed twice with LB medium to remove residual antibiotics and re-suspended in 100 μl of LB medium. *C. phytofermentans* was cultured anaerobically in GS2 glucose medium overnight to late log phase (0.9 OD_600_). Approximately 0.5 ml of the *C. phytofermentans* culture was then centrifuged and re-suspended in 100 μl of GS2 medium. The *E. coli* and *C. phytofermentans* were mixed, aliquoted on solid GS2 medium as a series of 10 μl spots, and allowed to mate overnight at 32°C in anaerobic jars. Parallel, mock matings of *C. phytofermentans* and *E. coli* conjugal donors lacking pQint3367 were conducted to ensure that erythromycin resistance in *C. phytofermentans* required transfer of pQint3367. After mating, the cells were re-suspended from the plates into 5 ml of liquid GS2 medium containing 40 μg ml^−1^ nalidixic acid and 10 μg ml^−1^ trimethoprim and were cultured anaerobically for 3 h. These cultures did not contain erythromycin to permit the *C. phytofermentans* to recover from the mating. The cultures were centrifuged, re-suspended in 50 μl of GS2, and plated on GS2 solid medium containing 40 μg ml^−1^ nalidixic acid, 10 μg ml^−1^ trimethoprim and 40 μg ml^−1^ erythromycin. The plates were incubated anaerobically for 6 days at 32°C. Erythromycin-resistant *C. phytofermentans* colonies were picked, transferred to liquid GS2 medium containing 200 μg ml^−1^ erythromycin and cultured anaerobically. Once cultures had grown to OD_600_ > 0.5, the absence of residual *E. coli* cells was confirmed by plating a 50 μl aliquot on solid LB medium and incubating aerobically at 37°C overnight.

### Southern blotting

To prepare a Southern blot, *C. phytofermentans* genomic DNA was isolated from 3 ml of cultures using a Qiagen Genomic Tip 20/G (Cat 10223). One microgram of genomic DNA was digested with HindIII, which does not cut in the intron insertion, and resolved by electrophoresis. The DNA was transferred to a nylon membrane (Perkin Elmer Genescreen, Cat NEF972001PK) and cross-linked using a Stratagene UV Stratalinker 2400. The blot was probed with a ^32^P-labelled intron PCR product that was amplified from pQint3367 with primers Ibs_3367 and Ebs1d_3367.

### qRT-PCR

Cultures of wild-type and AT02 *C. phytofermentans* strains were grown in GS2 medium containing either hemicellulose xylan, glucose or cellulose as a sole carbon source. Cells were collected by centrifugation in mid-log phase for glucose and hemicellulose cultures; samples were taken from cellulose cultures after 2 weeks of growth. RNA was extracted using Ribopure Bacteria Kit (Ambion AM1925) and residual DNA was removed using DNase I (Ambion AM2222). Total RNA yields were approximately 5 μg of RNA per ml of culture. One microgram RNA was reverse transcribed to single-stranded DNA using the Superscript First Strand cDNA Synthesis Kit (Invitrogen 11904018). Real-time PCR amplification was conducted using a MJ Research DNA Engine Opticon II machine by monitoring incorporation of SYBR green I (Invitrogen S7563). PCR primers were designed to amplify 100 bp products from each gene (see Supporting information S3 for primer sequences). Relative gene expression was quantified using the comparative C_T_ method (Schmittgen and Livak, 2008) with the 16S ribosomal sequence serving as the internal control gene. Expression levels represent the means of duplicate measurements taken from duplicate cultures (see Supporting information S3 for qRT-PCR data files).

### Mass spectrometry

Protein lysates were prepared by French press from cultures of wild-type *C. phytofermentans* grown in GS2 medium containing either glucose, hemicellulose xylan or filter paper cellulose as the sole carbon source. Proteins were precipitated by adding one-fourth volume of 100% (w/w) trichloroacetic acid in water, re-suspended in 1% SDS, 0.2 M NaOH and 10 mM DTT and incubated for 60 min at 37°C. Cysteine residues were then derivatized with iodoacetic acid (30 mM) at room temperature in the dark for 60 min. Five hundred micrograms of protein was resolved on 4–12% Bis-Tris SDS-PAGE gels (Invitrogen NP0335BOX). Gels were sliced into eight sections and proteins were in-gel digested as described in Shevchenko *et al.* (1996). The resulting peptides were subjected to C18 solid-phase extraction using Waters SepPak cartridges (Waters WAT054960). Purified peptides were analysed by microcapillary liquid chromatography tandem mass spectrometry using an in-house prepared 125-μm-ID C18 reversed-phase column and a hybrid linear quadrupole/FT-ICR mass spectrometer (LTQ FT, Thermo Scientific, Bremen, Germany) as described in Haas *et al.* (2006).

Acquired MS/MS spectra were searched against a concatentated target/decoy protein sequence database consisting of the predicted proteins from *C. phytofermentans* (NCBI NC_010001.faa), common contaminants such as trypsin and human keratins, and the reversed sequences of these proteins used as decoy component (Elias and Gygi, 2007). Database searches were performed using the SEQUEST algorithm (Eng *et al.*, 1994). Peptide assignments for each treatment were filtered to a protein false discovery rate of smaller than 1%. The number of peptides that could be uniquely mapped to *cphy3366–9* were defined based upon peptide sequence, charge state and the gel section from which they were identified. Peptide counts per protein represent the mean of duplicate cultures for each treatment.
